# Withdrawn as duplicate: Sparse dimensionality reduction for analyzing single-cell-resolved interactions

**DOI:** 10.1093/bioadv/vbaf230

**Published:** 2025-09-24

**Authors:** Niklas Brunn, Maren Hackenberg, Camila L Fullio, Tanja Vogel, Harald Binder

**Affiliations:** Institute of Medical Biometry and Statistics (IMBI), Faculty of Medicine and Medical Center, University of Freiburg, Freiburg im Breisgau 79104, Germany; Freiburg Center for Data Analysis, Modeling and AI (FDMAI), University of Freiburg, Freiburg im Breisgau 79104, Germany; Institute of Medical Biometry and Statistics (IMBI), Faculty of Medicine and Medical Center, University of Freiburg, Freiburg im Breisgau 79104, Germany; Freiburg Center for Data Analysis, Modeling and AI (FDMAI), University of Freiburg, Freiburg im Breisgau 79104, Germany; Institute of Anatomy and Cell Biology, Department Molecular Embryology, Faculty of Medicine, University of Freiburg, Freiburg im Breisgau 79104, Germany; Faculty of Biology, University of Freiburg, Freiburg im Breisgau 79104, Germany; Institute of Anatomy and Cell Biology, Department Molecular Embryology, Faculty of Medicine, University of Freiburg, Freiburg im Breisgau 79104, Germany; Institute of Medical Biometry and Statistics (IMBI), Faculty of Medicine and Medical Center, University of Freiburg, Freiburg im Breisgau 79104, Germany; Freiburg Center for Data Analysis, Modeling and AI (FDMAI), University of Freiburg, Freiburg im Breisgau 79104, Germany; Centre for Integrative Biological Signaling Studies (CIBSS), University of Freiburg, Freiburg im Breisgau 79104, Germany

This article has been withdrawn due to an error that caused the article to be duplicated. The definitive version of this article is published under https://doi.org/10.1093/bioadv/vbag047.

