# Publisher’s Note: Sparse dimensionality reduction for analyzing single-cell-resolved interactions

**DOI:** 10.1093/bioadv/vbag055

**Published:** 2026-03-25

This is a Publisher’s Note regarding: Niklas Brunn, Maren Hackenberg, Camila L Fullio, Tanja Vogel, Harald Binder, Sparse dimensionality reduction for analyzing single-cell-resolved interactions, *Bioinformatics Advances*, Volume 6, Issue 1, 2026, vbag047, https://doi.org/10.1093/bioadv/vbag047

Due to a production error, this paper was originally published in *Bioinformatics* on 24 September 2025 instead of *Bioinformatics Advances*. The incorrect version has been withdrawn, and the publisher apologises for the error.

